# Preliminary examination of disgust learning as a potential OCD endophenotype

**DOI:** 10.1016/j.jad.2026.121619

**Published:** 2026-03-12

**Authors:** Sarah C. Jessup, David H. Zald, Bunmi O. Olatunji

**Affiliations:** a Vanderbilt University, United States of America; b Rutgers University, United States of America

**Keywords:** OCD, Disgust conditioning, Disgust proneness, Endophenotype

## Abstract

Endophenotypes (intermediate phenotypes) are traits hypothesized to represent genetic risk for disorders such as OCD. However, it is unclear if disgust conditioning abnormalities may function as an OCD endophenotype. The present study sought to examine the extent which disgust conditioning abnormalities characterize OCD in a manner that is consistent with an endophenotype. Participants with OCD (*n* = 30), first-degree relatives (*n* = 27), and unrelated age-matched healthy controls (*n* = 25) completed a disgust conditioning task in which one neutral food item (conditioned stimulus; CS+) was followed by disgusting videos of individuals vomiting (unconditioned stimulus; US) and another neutral food item (CS−) was not reinforced with the disgusting video. Following this acquisition phase, there was an extinction phase in which both CSs were presented unreinforced. As predicted, the CS+ was rated as significantly more disgusting than the CS− after two blocks of acquisition and this pattern was eliminated after extinction. Although no group differences emerged as a function of the CS+ compared to the CS−, the OCD group reported statistically significantly higher disgust ratings to both CSs during acquisition (but not at habituation or extinction) than relatives and controls. However, relatives and controls did not significantly differ from each other. Additionally, disgust proneness explained the group difference between those with and without OCD in disgust responding to both CSs at acquisition. These findings suggest a readiness for disgust conditioning in OCD that may be the product of heightened disgust proneness but provide no evidence that it functions as an endophenotype.

## 1. Introduction

Obsessive-compulsive disorder (OCD) is characterized by recurrent obsessions and/or compulsions that interfere with functioning (*DSM-5*; American Psychiatric Association [APA], 2013). Obsessions consist of intrusive, repetitive thoughts, images, or impulses whereas compulsions are purposeful, repetitive behaviors or rituals performed to relieve distress associated with obsessions. Although the etiology of OCD is complex, there has been growing interest in the identification of endophenotypes (intermediate phenotypes), or objective, heritable, quantitative traits that reflect genetic risk for the disorder (Chamberlain and Menzies, 2009). For example, Menzies et al. (2007) found that structural variation in large-scale brain systems related to motor inhibitory control may mediate genetic risk for OCD, representing the first evidence for a neurocognitive OCD endophenotype. Research also suggests that presupplementary motor area hyperactivity may be a neurocognitive endophenotype of OCD that is related to inefficient neural processing within the presupplementary motor area (de Wit et al., 2012). Other promising OCD endophenotypes include abnormal EEG patterns associated with error processing, deficits on tests of executive functioning, and particular types of strongly held dysfunctional beliefs (Taylor, 2012). The identification of endophenotypes of this sort may inform ongoing efforts to more precisely clarify the etiological and diagnostic status of OCD (Vaghi, 2021).

Although the examination of endophenotypes in OCD has largely focused on executive processing deficits when comparing OCD patients with their first-degree relatives (de Wit et al., 2012; van Velzen et al., 2015), there has also been recent interest on emotional processing deficits as potential endophenotypes (Thorsen et al., 2019). Within the emotion domain, the unique role of disgust has emerged as a particular focus in OCD. Indeed, Shapira et al. (2003) found that whereas brain activation during a threat-inducing task in OCD patients was similar to healthy controls, the pattern of activation during the disgust-inducing task was significantly different, including greater increases in the right insula, parahippocampal region, and inferior frontal sites for those with OCD. A growing body of research now suggests that those with OCD experience disgust more frequently and intensely than those without OCD, a characteristic that is often referred to as ‘disgust proneness’ (Olatunji et al., 2017a). Disgust proneness reflects how susceptible a person is to feeling disgust and how unpleasant they find that experience. Longitudinal research suggests that disgust proneness is a largely time-invariant or trait-like personality characteristic that confers risk for OCD (Olatunji et al., 2020). Although the origin of excessive disgust proneness is likely complex, there is some evidence suggesting that this stable trait may emerge due to an interaction of genetics (Olatunji et al., 2019; Sherlock et al., 2016) and childhood socializing experiences where disgust responses are modeled excessively (Stevenson et al., 2010). This gene X environment interaction that shapes the development of disgust proneness may also confer risk for the development of OCD.

Evidence implicating disgust in OCD has been observed across multiple levels of analysis (Cisler et al., 2009; Olatunji et al., 2017a). This body of research also suggests that those whose OCD is characterized primarily by contamination obsessions and washing compulsions are most likely to experience disgust more frequently and intensely (Tolin et al., 2006). However, Olatunji et al. (2011a, 2011b) found that disgust proneness predicted an OCD symptom latent factor that consisted of washing concerns, checking and doubting, obsessing, neutralizing, ordering, and hoarding even when controlling for negative affect. Disgust proneness is also significantly associated with OCD that is primarily characterized by scrupulosity (Inozu et al., 2017; Olatunji et al., 2005) and sexual orientation concerns (Ching et al., 2021). These findings suggest that disgust proneness is not unique to contamination-based OCD and the tendency towards behavioral inhibition to avoid punishment and non-reward may partially account for the association between disgust proneness and OCD subtypes that are not characterized by contamination fear (Olatunji et al., 2009).

Heightened disgust proneness may also be observed in OCD from a conditioning framework where disgust associations may form more easily (heightened disgust learning) and persist in the absence of a CS/US contingency (impaired disgust extinction). Studies on disgust conditioning have examined differences in a conditioned stimulus (CS; a neutral word or face) that was never paired with a disgusting US (CS−) and a CS that was always paired with a disgusting US (CS+). Such studies have consistently shown that during acquisition, the CS+ elicited stronger subjective disgust than the CS− (Olatunji et al., 2007; Olatunji et al., 2013). A recent meta-analysis directly comparing the degree of extinction of conditioned disgust and conditioned fear in laboratory paradigms found that learned disgust was significantly more resistant to extinction than fear (Mitchell et al., 2024). In fact, there is strong evidence that conditioned disgust can easily generalize (Berg et al., 2021; Olatunji and Tomarken, 2023). Although few studies have examined the neural basis of disgust conditioning, preliminary research using functional magnetic resonance imaging (fMRI) found activation in the cingulate cortex, nucleus accumbens, orbitofrontal cortex, and the occipital cortex during disgust conditioning (Klucken et al., 2012). Experimental studies have also observed meaningful distinctions between fear and disgust conditioning. For example, conditioned disgust responses are more resistant to extinction than conditioned fear responses (Mason and Richardson, 2010; Olatunji et al., 2007). Furthermore, Klucken et al. (2012) found that conditioned disgust is associated with greater insula activation than fear conditioning, which is consistent with a proposed heightened interoceptive sensitivity that is more pronounced with disgust.

Although no study to date has examined disgust conditioning among patients with OCD, Armstrong and Olatunji (2017) found that undergraduate students with high (HCC) and low (LCC) contamination concerns and washing compulsions did not differ from one another in discriminant responding to the CSs following disgust acquisition. However, following extinction, the HCC group reported less reduction in their expectancy of the disgusting US following the CS+, and greater disgust to the CS+, compared to the LCC group. Novara et al. (2021) more recently examined HCC and LCC participants that completed a differential associative learning task in which neutral images were followed by disgusting images (CS+) or not (CS−). Following this acquisition phase, there was a counter-conditioning procedure in which CS+ was followed by a pleasant unconditioned stimulus while CS− remained unreinforced. Following counter-conditioning, both groups reported significantly less disgust for the CS+ and reduction in disgust for the CS+ was lower in the HCC group than in the LCC group. Although available analogue studies suggest that a Pavlovian disgust conditioning approach may inform etiological and treatment models of contamination concerns commonly observed in OCD, the extent to which heightened disgust conditioning can be considered a candidate endophenotype for OCD remains unclear. Endophenotypes are conceptualized as measurable intermediary variables lying somewhere in a causal chain linking genes to a given disorder (Taylor, 2012) and can be detected both in patients and in unaffected relatives of individuals with the disorder (Cannon and Keller, 2006). To our knowledge, no study to date has examined disgust conditioning as an OCD endophenotype that may be part of the causal link of the disorder by comparing disgust conditioning between those with OCD, their unaffected first-degree relatives, and healthy controls.

To determine whether disgust conditioning abnormalities may be characteristic of OCD, a Pavlovian disgust conditioning procedure was employed in the present study in which one neutral food item (conditioned stimulus; CS+) was followed by disgusting videos of individuals vomiting (unconditioned stimulus; US) and another neutral food item (CS–) was not reinforced with the disgusting video. Following this acquisition phase, there was an extinction phase in which both CSs were presented unreinforced. Theoretical models suggest that at its core, disgust is a food-related emotion that facilitates revulsion at the prospect of oral incorporation of offensive foods (Rozin and Fallon, 1987). Accordingly, use of CSs that reflect the food-based function of disgust may offer more sensitivity and specificity especially when paired with an unlearned response to foods that occurs naturally. This is an important consideration given that CS − US combinations that ‘belong’ together are more easily learned and consequently may more easily result in the transfer of the conditioned disgust response to stimuli that resemble the original CS (i.e., Garcia and Koelling, 1966; Hamm et al., 1989).

Another goal of the present study was to employ a disgust conditioning paradigm where the neutral CS ‘belongs’ to a contingently occurring disgust-related US to examine disgust learning as a potential OCD endophenotype. It was predicted that the repeated pairing of a food item (CS+) with video presentation of an individual vomiting (US) would lead participants to evaluate the food item as more disgusting than a food item (CS−) that is not paired with vomit. For this hypothesis, we expected disgust ratings of the CS+, but not the CS−, to exhibit significant changes across conditioning phases to match a classic quadratic profile with ratings incrementally increasing from habituation to the first phase of acquisition to the second phase of acquisition and then decreasing after extinction. Consistent with an endophenotype model, heightened disgust learning during acquisition and extinction was predicted to be observed more predominantly in OCD patients and relatives compared to controls. We also tested the hypothesis, suggested by extant research (i.e., Armstrong and Olatunji, 2017), that heightened disgust learning that may characterize those with OCD may be explained by elevated levels of disgust proneness.

## 2. Method

### 2.1. Participants

A total of 82 individuals participated in the study: 30 participants with OCD, 27 first-degree relatives, and 25 healthy controls. Participants ranged from 15 to 64 years old (M = 32.21, SD = 13.61). The sample was 63.4% female (*n* = 52) and 8.5% identified as Hispanic/Latino (*n* = 7). The racial composition was as follows: 82.9% White (*n* = 68), 14.6% Black or African American (*n* = 12), 1.2% Asian or Pacific Islander (*n* = 1), and 1.2% Biracial (*n* = 1). Approximately two thirds of participants (*n* = 53, 60.2%) were single and had completed at least a college degree (*n* = 58, 65.9%). Among those with OCD, 30% presented with comorbid generalized anxiety disorder, 16.7% with panic disorder, 16.7% with social anxiety disorder, 6.7% with dysthymia, and 3.3% with major depressive disorder.

Individuals with OCD were recruited from flyers placed at local clinics, private practices, community centers, and hospitals. Interested individuals completed a phone screen with a trained post-baccalaureate research assistant. During the phone screen, the research assistant administered the OCD module of the MINI International Neuropsychiatric Interview (MINI; Sheehan et al., 1998), the Yale-Brown Obsessive-Compulsive Scale (Y-BOCS; Goodman et al., 1989), assessed for current medications, the presence of a local, first-degree relative, and rule-out conditions. To be considered eligible, an OCD participant was required to meet criteria on the OCD module of the MINI, endorse at least mild to moderate symptoms on the Y-BOCS, and identify a local, first-degree relative without OCD who was also willing to participate in the study. Given that the role of disgust is most prominent in contamination-based OCD (e.g., Bhikram et al., 2017), OCD participants also had to endorse some obsessions related to contamination on the MINI to be considered eligible (although contamination did not need to be the primary obsession). First-degree relatives were considered eligible if they did not meet criteria for OCD on the MINI.

Age-matched healthy controls were recruited from flyers placed around the university and through ResearchMatch, a national health volunteer registry that was created by several academic institutions and supported by the U.S. National Institutes of Health. Healthy controls were considered eligible if they did not meet criteria for any disorder on the MINI. Exclusionary criteria, which were the same for all three conditions, included current diagnoses of traumatic brain injury, bipolar disorder, substance abuse, pervasive developmental disorder, and a history of central nervous system disease.

### 2.2. Measures

#### Yale-Brown Obsessive-Compulsive Scale (Y-BOCS; Goodman et al., 1989).

The Y-BOCS is a semi-structured interview to briefly measure the following five parameters of obsessions (items 1–5) and compulsions (items 6–10): time occupied/frequency, interference, distress, resistance, and perceived control. Participants are asked to rate each item on a 5-point Likert scale from 0 (*no symptoms*) to 5 (*severe symptoms*). Obsession and compulsion scores (items 1–10) are summed to yield a total score. The Y-BOCS has been shown to yield satisfactory validity and reliability (Goodman et al., 1989).

#### Obsessive-Compulsive Inventory- Revised (OCI-R; Foa et al., 2002).

The OCI-R is an 18-item self-report measure that assesses OCD symptoms. Participants are asked to rate their experiences in the past month on a 5-point Likert scale ranging from 0 (*not at all*) to 4 (*very much*). The OCI-R is comprised of six dimensional subscales, including washing, checking, ordering, obsessing, hoarding, and neutralizing, which are summed to yield a total score. The OCI-R demonstrated excellent internal consistency in the current sample (α = 0.94).

#### The Disgust Propensity and Sensitivity Scale- Revised (DPSS-R; van Overveld et al., 2006).

The DPSS-R is a 16-item self-report questionnaire that assesses the frequency of experiencing disgust (i.e., propensity) and the emotional impact of those symptoms (i.e., sensitivity). Participants rate their agreement with each item using a scale of 0 (*never*) to 4 (*always*). The DPSS-R was found to have excellent internal consistency in the current sample (α = 0.92).

#### The State-Trait Anxiety Inventory, Trait Scale, Form Y (STAI-T; Spielberger et al., 1983).

The STAI-T is a 20-item self-report questionnaire that assesses the enduring or chronic experience of anxiety. Participants rate how they generally feel on a scale from 1 (*almost never*) to 4 (*almost always*) on statements such as “I feel nervous and restless” and “I am ‘calm, cool, and collected.’” The STAI-T was found to have excellent internal consistency in the current sample (α = 0.95).

### 2.3. Materials

#### 2.3.1. Conditioned stimulus (CS)

The CSs consisted of two types of neutral food items (i.e., cheese and pita bread) that have been used in previous research on disgust conditioning (Borg et al., 2016; Olatunji, 2020; Olatunji et al., 2017b). The two food items were counterbalanced (i.e., both items were equally presented as the CS+ and CS−). These CSs have been validated with regards to being equivalently neutral (Borg et al., 2016).

#### 2.3.2. Unconditioned stimulus (US)

The USs consisted of 4 different sound-attenuated video clips depicting individuals vomiting. The videos depicting people vomiting have been shown in previous research to strongly evoke the desired emotion of disgust, but not fear (Armstrong et al., 2014). As a neutral outcome, sound-attenuated films depicting waterfall scenes were also used (NS). Multiple USs of people vomiting were included in the present study to prevent habituation.

#### 2.3.3. Self-report affective assessment of CS

Following each conditioning phase, participants rated how disgusted the CS+ and CS− made them feel. A button box attached to participants’ dominant hand was used for ratings during the conditioning task that was presented using *E*-Prime 3.0 software. The button box contains five buttons (one for each digit) that correspond to ratings on the participants’ screen. Participants rated their disgust by pushing the corresponding button ranging from *not at all disgusting* to *very disgusting*.

### 2.4. Procedure

The university’s Institutional Review Board approved the study and all participants provided informed consent. For participants under 18 years old (*n* = 5; 5.7%), the individual provided informed assent and their legal guardian who was also participating in the study provided consent. For all participants, a trained post-baccalaureate research assistant^1^ assessed the presence/absence of an OCD diagnosis and comorbid diagnoses using the MINI (Sheehan et al., 1998), a structured diagnostic tool that assesses 17 disorders consistent with the fourth edition of the Diagnostic and Statistical Manual of Mental Disorders (DSM-IV; APA, 1994) in Session 1. All participants were then seated at a laboratory computer and completed the self-report measures.

**Fig. 1.**
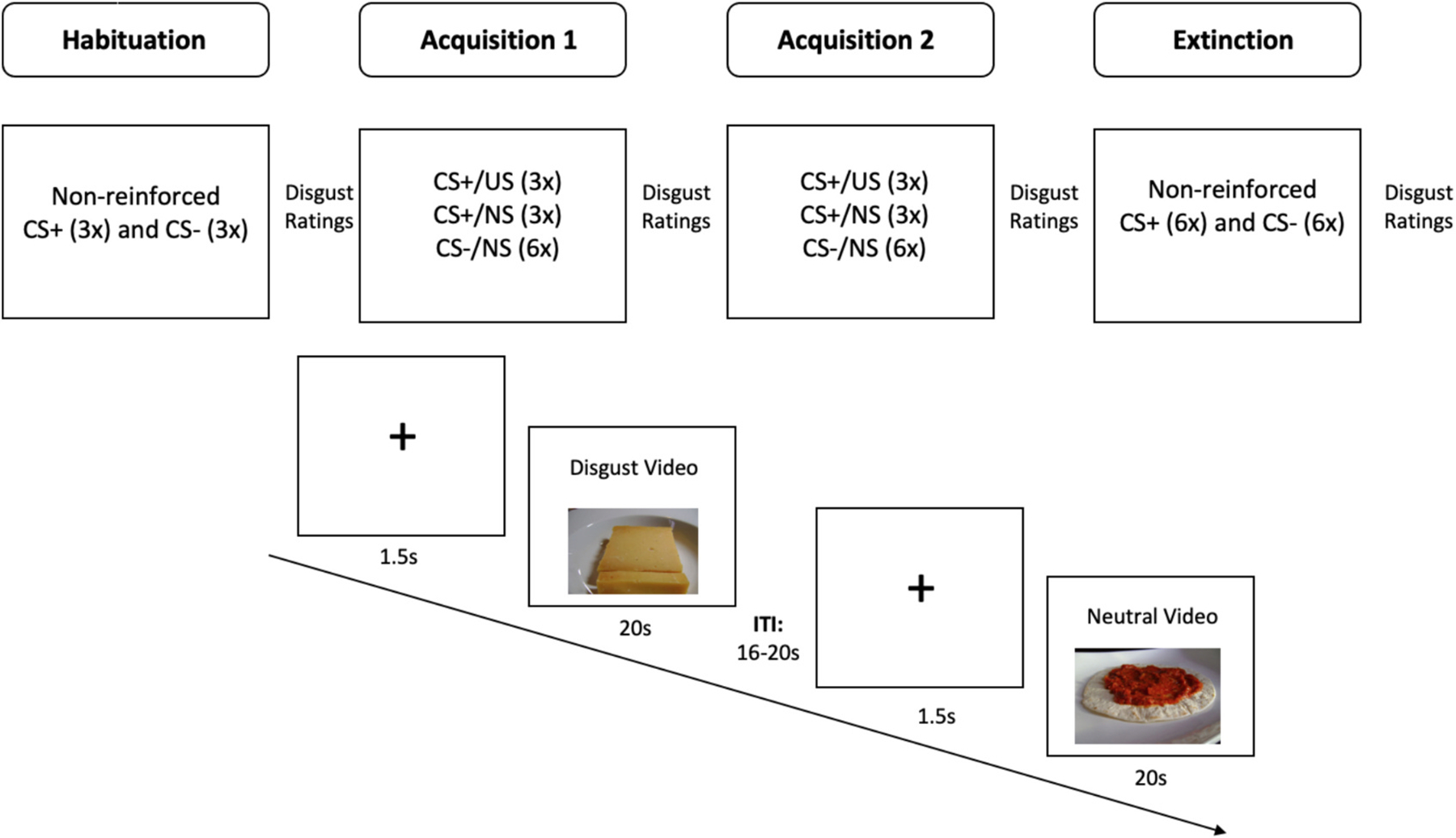
Overview of disgust conditioning task.

Participants returned for session two to complete the disgust conditioning task. The task consisted of the following phases: habituation, acquisition 1, acquisition 2, and extinction. During the habituation phase, participants viewed a non-reinforced presentation (15 s) of each CS in random order. The CSs appeared in the lower third of the screen and participants were instructed to look directly at the CS for the entire duration. CSs were preceded by a fixation cross (1.5 s) and followed by an inter-trial interval (ITI) that varied randomly between 16 s and 20 s. During acquisition, the CSs were presented for 20 s in the lower third of the screen. After the first 5 s of presentation, a video (i.e., US or NS) began playing in the top third of the screen and remained for the final 15 s of the CS presentation. Participants were instructed to look directly at the CS until the video began, and then to watch the video. As with habituation, CSs were preceded by a fixation cross (1.5 s) and followed by an ITI that varied randomly between 16 s and 20 s. In total, the CS+ was presented with a video 6 times; 3 trials of the CS+ paired with a US video and 3 trials of the CS+ paired with an NS video. That is, the CS+ was reinforced only 50% of the time. A 50% reinforcement of the CS+ was used given research showing that continuous pairing suffered from rapid extinction compared with a conditioning procedure that used partial pairing (Grady et al., 2016). The CS− was paired with the NS videos 6 times. In total, 6 CS+ trials and 6 CS− trials were presented in random order. The acquisition phase was then repeated. During extinction, participants viewed the CSs without the accompanying video. There were 6 trials of CS+ and 6 trials of CS− (see Fig. 1 for a schematic of the disgust conditioning task).

### 2.5. Data analytic overview

A repeated-measures multivariate analysis of variance (MANOVA) was used to assess group differences for the CS (CS+, CS−) for the four phases of conditioning: habituation, acquisition 1, acquisition 2, extinction. Participants with incomplete conditioning data in one or more conditioning phases were excluded (*n* = 7; 3 OCD participants, 2 relatives, 2 controls). As part of the MANOVA procedure, simple tests of within-subject contrasts were used to identify the pattern (i.e., linear, quadratic, or cubic) and significance of the conditioning procedure. Quadratic trend analyses were particularly important for testing the shape of disgust learning given multiple phases of acquisition, with the a priori hypothesis that learning pattern for OCD patients would more robustly reflect a quadratic function compared to relatives and controls.

## 3. Results

### 3.1. Group differences in symptom measures

A one-way Analysis of Variance (ANOVA) was conducted to examine group differences in scores on the OCI-R. The one-way ANOVA was significant [*F*(2, 79) = 32.82, *p* < .001] and post-hoc Fisher’s LSD tests revealed that compared to the first-degree relative and control groups, the OCD group reported more symptoms on the OCI-R (*p*s < 0.001). However, the first-degree relative and control groups did not significantly differ from one another on OCI-R scores (*p* > .10). A one-way ANOVA was also significant for the DPSS-R [*F*(2,79) = 17.41, *p* < .001] and STAI-T total scores [*F*(2,79) = 23.62, *p* < .001]. Follow-up Fisher’s LSD tests revealed that compared to the first-degree relative and control groups, the OCD group reported greater scores on the DPSS-R and STAI-T (*p*s < 0.001). However, the first-degree relative and control groups did not significantly differ on the DPSS-R or STAI-T (*p*s > 0.10). Means and standard deviations for all measures are provided in Table 1.

Similarly, chi square tests were conducted to examine potential group differences in comorbid anxiety/depression rates and medication use. Compared to first-degree relative and control conditions, a greater number of individuals in the OCD condition met criteria for a co-morbid anxiety disorder, χ^2^ = 30.71, *p* < .001, and were taking medication, χ^2^ = 82.00, *p* < .001. The first-degree relatives and healthy controls did not significantly differ from one another (all *p*s > 0.10). The three groups did not significantly differ in rates of comorbid depression (*p* > .10).

### 3.2. Group differences in disgust conditioning

A 2 (CS: CS+, CS−) × 4 (Phase: Habituation, Acquisition 1, Acquisition 2, Extinction) × 3 (Group: OCD, Relative, Control) mixed-effects ANOVA was conducted to examine group differences in disgust ratings throughout the conditioning paradigm (see Table 2). The tests of within subjects effects yielded the predicted significant linear main effect of CS [*F*(1,58) = 5.75, *p* = .02, $\eta_{\text{p}}^{2}=0.09$] and a significant quadratic main effect of phase [*F*(1,58) = 6.33, *p* = .01, $\eta_{\text{p}}^{2}=0.09$]. The test of between subjects effects also revealed a significant main effect of group [*F*(1,58) = 5.84, *p* < .01, $\eta_{\text{p}}^{2}=0.16$] reflecting overall higher ratings of disgust in the OCD group. A significant quadratic CS X phase interaction [*F*(1,58) = 7.23, *p* < .01, $\eta_{\text{p}}^{2}=0.11$] and a marginally significant quadratic group X phase interaction [*F*(2,58) = 2.90, *p* = .06, $\eta_{\text{p}}^{2}=0.09$] were also observed. However, the predicted quadratic CS X phase X group interaction was not significant [*F*(2,58) = 0.57, *p* = .56, $\eta_{\text{p}}^{2}=0.00$].

**Fig. 2.**
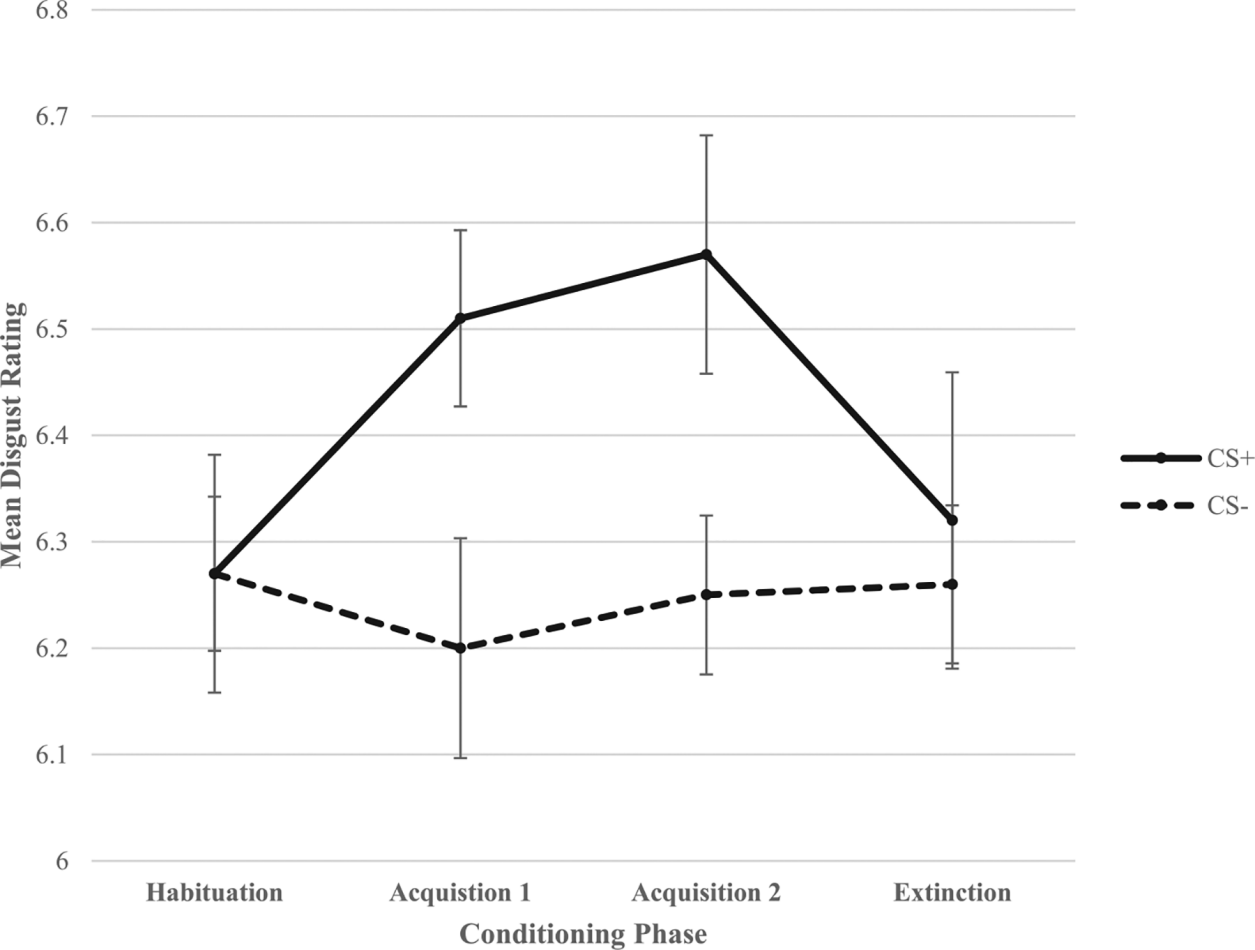
Mean disgust ratings for the conditioned stimuli (CS) across the four phases of conditioning in the full sample. Error bars: ±1 standard error.

**Fig. 3.**
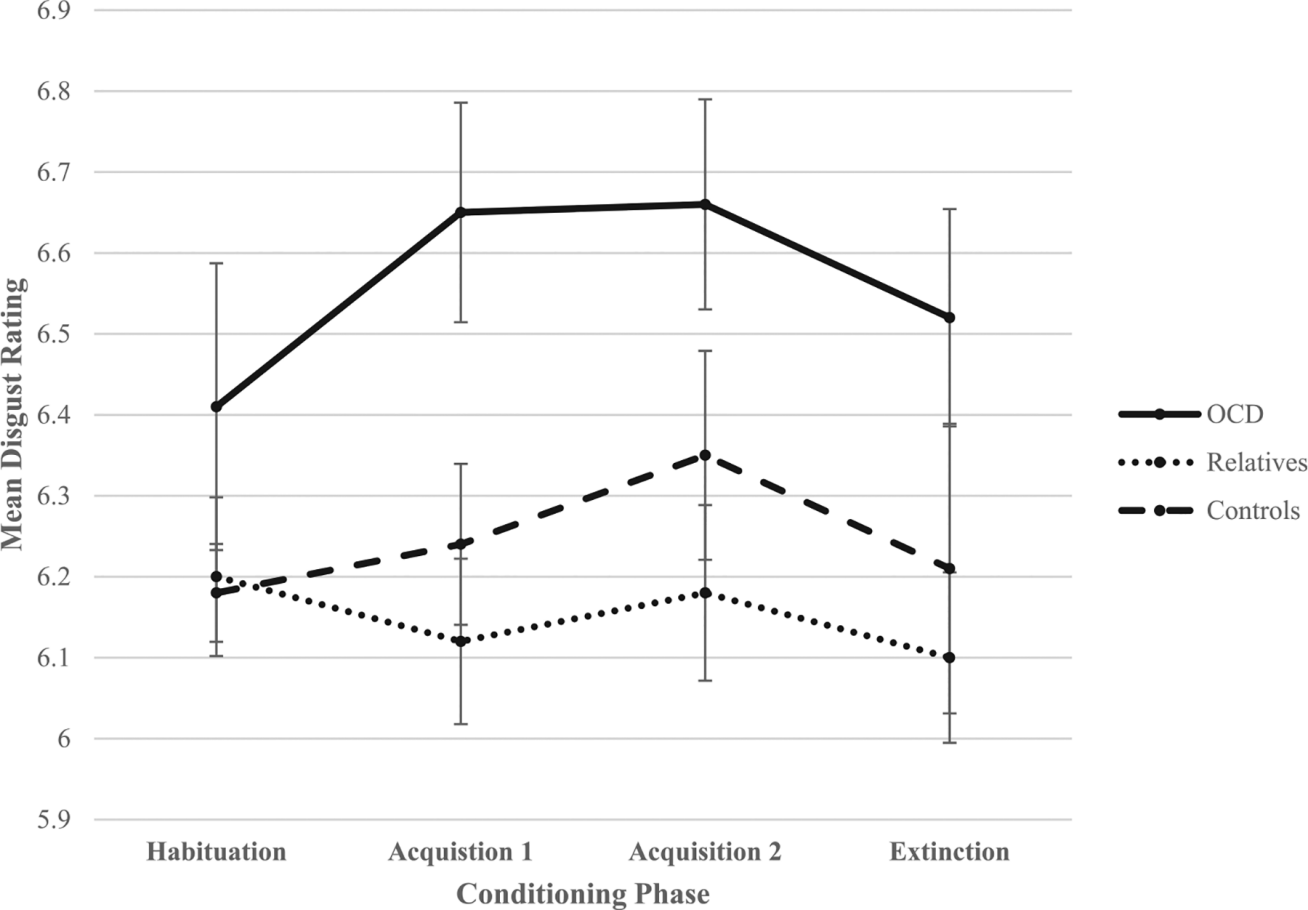
Mean disgust ratings for both conditioned stimuli across the phases of conditioning. Error bars: ±1 standard error.

As shown in Fig. 2, the significant quadratic CS X phase interaction shows that although there are no significant differences in disgust ratings of the CS+ and CS− at habituation and extinction (*p*s > 0.10), participants rated the CS+ as significantly more disgusting than the CS− at acquisition 1, *t*(78) = 2.557, *p* = .013, and acquisition 2, *t*(78) = 2.70, *p* = .008. A probe of the marginally significant quadratic group X phase interaction which examines disgust ratings collapsing across both CSs revealed no significant group differences at habituation (*p* = .38) or extinction (*p* = .105). However, significant group differences were observed at acquisition 1, *F*(2,76) = 5.85, *p* = .004, and acquisition 2, *F*(2,76) = 3.90, *p* = .024 (see Fig. 3). LSD post hoc tests revealed that the OCD group reported significantly higher disgust ratings of the CS than relatives (*p* = .002) and controls (*p* = .015) at acquisition 1. However, relatives and controls did not significantly differ in disgust ratings of the CS at acquisition 1 (*p* = .48). The OCD group also reported significantly higher disgust ratings of the CS than relatives (*p* = .008) and marginally higher disgust ratings of the CS than controls (*p* = .08) at acquisition 2. However, relatives and controls did not significantly differ in disgust ratings of the CS at acquisition 2 (*p* = .35).

### 3.3. The role of disgust proneness in disgust conditioning

**Fig. 4.**
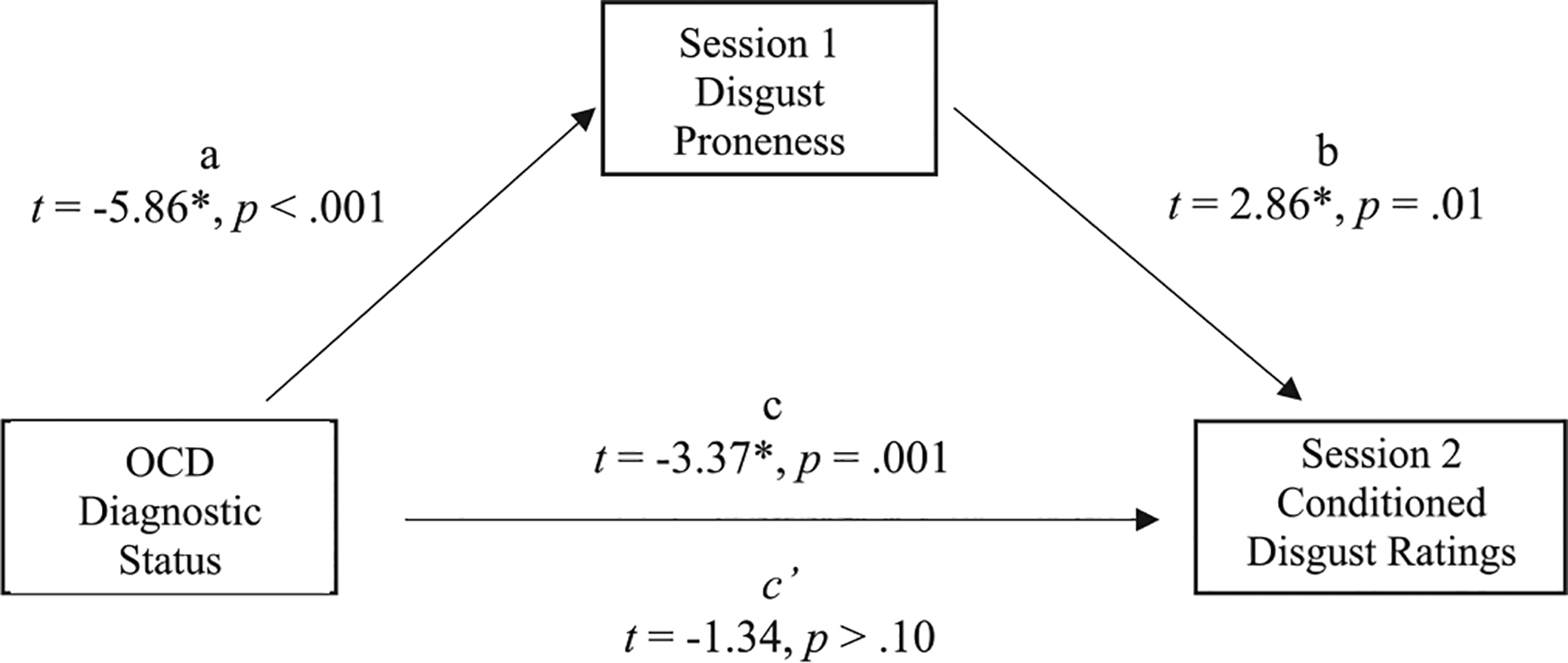
Disgust proneness at session 1 accounts for the association between diagnostic status (OCD vs. no OCD) and conditioned disgust ratings during acquisition at session 2. Path c in the model represents the total effect and path c’ represents the direct effect.

The extent to which disgust proneness explains the relationship between diagnostic status (OCD vs. no OCD) and higher disgust ratings to both CSs during acquisition was also examined. The first-degree relative and control conditions were collapsed into one group for a comparison of individuals with versus without OCD. Similarly, disgust ratings to both CSs were collapsed across acquisitioning trials. The DPSS-R total score was used as the measure of disgust proneness. Hayes’ (2013) PROCESS macro and 95% bootstrap confidence intervals were used to determine whether the indirect effect was significant. If zero does not lie within the generated 95% confidence interval, one can reasonably conclude that the indirect effect significantly differs from zero and thus, disgust proneness explains the relationship between diagnostic status and conditioned disgust responses. A 95% bootstrap confidence interval revealed the true indirect effect for disgust proneness was estimated to lie between −0.50 and − 0.03 (Effect = −0.23, SE = 0.12). This 95% bootstrap confidence interval does not contain zero and after accounting for disgust proneness, the direct effect of diagnostic status on conditioned disgust responses was no longer significant (*p* > .10). Thus, as depicted in Fig. 4, it can be reasonably concluded that disgust proneness fully explains the relationship between diagnostic status and conditioned disgust responses during acquisition.

## 4. Discussion

A major aim of the present study was to examine the extent to which disgust conditioning abnormalities may be characteristic of OCD. The findings showed that disgust ratings of the CS+, but not the CS−, significantly increased across conditioning phases to match the classic quadratic profile with ratings incrementally increasing from habituation to the first phase of acquisition to the second phase of acquisition and then decreasing after extinction. This finding was observed with the food item (CS+) paired with vomit at a reinforcement rate of 50%. This finding that the repeated pairing of a neutral stimulus with an inherently disgusting US results in the neutral stimulus becoming a CS that also induces the experience of disgust is consistent with previous research (Mason and Richardson, 2010; Olatunji et al., 2007). This form of learning may reflect a “hedonic shift” in the CS+ where the food item itself is perceived as disgusting (De Houwer et al., 2001). Although individual differences in the perceived strength of the co-occurrence of a CS+ and a disgust-relevant US may have implications for conceptualizing symptoms of OCD, no significant group differences emerged as a function of the CS+ compared to the CS−.

The present study did find that the OCD group reported significantly higher disgust ratings to both CSs during acquisition, but not at habituation or extinction, compared to relatives and controls. However, relatives and controls did not significantly differ from each other. This finding does converge with prior research suggesting that disgust conditioning abnormalities may be characteristic of OCD (Novara et al., 2021). Furthermore, a comprehensive review of the literature suggests that disgust conditioning abnormalities may be especially relevant for contamination subtype of OCD (Ludvik et al., 2015). In fact, Armstrong and Olatunji (2017) suggest that excessive contamination concerns observed in OCD may be related to difficulty inhibiting conditioned disgust responses. However, the “hedonic shift” in disgust during acquisition in the OCD group compared to relatives and controls was not specific to the CS+. Rather, the pattern of an “hedonic shift” in disgust during acquisition in the OCD group was observed for the CS+ and CS−. Although one interpretation is that this may reflect relatively poorer discrimination between learned disgust-cues (CS+) and learned safety-cues (CS−), a more likely interpretation is that it reflects a general propensity to respond with disgust among those with OCD.

A consistent finding in the literature is that OCD is uniquely characterized by heightened disgust proneness (Olatunji et al., 2011b; Olatunji et al., 2017a, 2017b). Prior research has also shown that individual differences in disgust proneness predict facets of conditioned disgust responding (Armstrong et al., 2014; Olatunji et al., 2013; Olatunji and Tomarken, 2023). Furthermore, Armstrong and Olatunji (2017) found that disgust proneness mediated differences between HCC and LCC participants in disgust responding to the CS+ at acquisition and extinction. Accordingly, the present study examined the extent to which group differences in conditioned disgust responding were explained by disgust proneness. The findings showed that disgust proneness (assessed at session 1) fully explained the group differences between those with OCD and those without in disgust responding to both CSs at acquisition (assessed at session 2 1 week later). This pattern of findings may have important implications for how disgust proneness may be conceptualized in etiological models of contamination-based OCD. This finding may speak to potential causal effects of disgust proneness on more generalized conditioned disgust responses among those with OCD that warrants investigation in future research. This pattern of associations may contribute to poor contagion-safety discrimination in OCD and subsequently more pervasive behavioral avoidance of ambiguous sources of contamination. A general conditioned disgust response that stems from disgust proneness in OCD may also function to maintain the disorder by motivating the unnecessary use of safety behaviors, such as washing or cleaning behaviors, that prevent corrective learning.

There has been increased interest in intermediate vulnerability markers that may confer risk for OCD (Chamberlain and Menzies, 2009). Such endophenotypes are objective traits that may represent genetic risk for OCD and the identification of these traits may inform etiological models. An endophenotype should be observed in unaffected family members at a higher rate than in the general population (Hasler et al., 2004). A candidate endophenotype may be observed across multiple levels of analysis including biochemical, neurophysiological, neuroanatomical, cognitive, or other processes, including those derived from self-report measures (Cannon and Keller, 2006; Gottesman and Gould, 2003). Given the growing body of research implicating disgust in OCD (Knowles et al., 2018), it may be that disgust relevant processes are potential candidate endophenotypes. Although the present study did find that the OCD group reported greater disgust proneness than the first-degree relative and control groups, the first-degree relative and control groups did not significantly differ from one another on DPSS-R scores. This finding is inconsistent with the view of disgust proneness as an endophenotype given that it was not elevated in patients with OCD *and* their first-degree relatives compared to controls.

Twin modeling research has shown that disgust proneness is partially heritable (Olatunji et al., 2019; Sherlock et al., 2016) and multivariate twin modeling has also found that the significant association between disgust proneness and contamination sensitivity commonly observed in OCD reflects overlapping genetic (54%) influences (Tybur et al., 2020). Despite the heritability of disgust proneness and the demonstrated link with symptoms of OCD, the present study suggests that disgust proneness may not satisfy the requirements for an endophenotype given that it was not significantly elevated within family members of those with OCD. Employing an endophenotype approach to disgust proneness in OCD is hypothesized to facilitate the identification of susceptibility genes since this approach aims to deconstruct the clinical phenotype into biological variables that are hypothetically more proximal to genetic effects. Although the dopamine receptor D4 (DRD4) and catechol-*O*-methyltransferase (COMT) genes have been implicated in disgust proneness (Kang et al., 2010) and the same genes have been implicated in OCD (Nestadt et al., 2010), it is unclear if disgust proneness is reflective of a biomarker for OCD in part because of shared underlying genetic influences. In addition to being associated with illness in the population, another consideration for an endophenotype is that it should be primarily state-independent (Murray and Lopez, 1997). A recent study examining the extent to which disgust proneness reflects a time-varying (TV) or state-like factor versus a time-invariant (TI) or trait-like personality characteristic found that although estimates of TI factor variance and TV factor variance were both significant, the proportion of TI factor variance was substantially and significantly greater than the amount of TV factor variance (Olatunji et al., 2020). This suggests that while disgust proneness is indeed primarily state-independent, it may not function as a psychological endophenotype for OCD.

Another aim of the present study was to examine the extent to which disgust conditioning abnormalities may also function as an OCD endophenotype. Although the present study did find that the OCD group reported greater disgust to both CSs at acquisition than the first-degree relative and control groups, the first-degree relative and control groups did not significantly differ from one another. This finding is also inconsistent with the view that disgust conditioning abnormalities may function as an OCD endophenotype given that such abnormalities were not elevated in patients *and* their first-degree relatives compared to controls. However, this does raise a larger question as to the extent to which conditioning abnormalities are useful models for examining endophenotypes. A recent study did find that amygdala engagement in response to conditioned faces with a social-evaluative meaning does qualify as a neurobiological candidate endophenotype of social anxiety (Bas-Hoogendam et al., 2020). However, prior research has shown that experimentally-derived fear conditioning measures share only a small portion of the genetic factors underlying individual differences in subjective fears (Hettema et al., 2008). This suggests that researchers should exercise caution against relying too heavily on conditioning abnormalities as an endophenotype for genetic studies of anxiety and related disorders.

Although the present study found no evidence supporting the view that disgust conditioning abnormalities may be conceptualized as an OCD endophenotype, there was some evidence that such abnormalities may be characteristic of OCD. However, study limitations must be considered when interpreting these findings. One important limitation is that the group comparisons were made with relatively small samples suggesting that the present study was likely underpowered to detect the predicted effects, similar to other studies that used small samples (Lissek et al., 2014). It is possible that type two errors occurred, such as in instances where null effects were reported for first-degree relative and healthy control comparisons. Similarly, the relatively small number of conditioning trials may have limited our ability to detect the predicted pattern of group differences. This is an important consideration given prior research suggesting that affective learning requires more trials to reflect a contingency change than do contingency judgments (Lipp and Purkis, 2006). This consideration would also support the integration of valence assessments across several trials in future research rather than the retrospective disgust ratings optioned in the present study. Future research with larger samples that employs a disgust conditioning task with more trials may provide more sensitivity for determining the extent to which disgust conditioning and extinction abnormalities may be an OCD endophenotype. Given that disgust conditioning and disgust proneness apply to a broad range of stimuli beyond contamination-specific stimuli, it is also possible that the use of contamination images and videos (i.e., vomit) partially limited the ability to detect endophenotypic group differences and neglects other disgust domains (e.g., moral) that are relevant to OCD. Thus, future research on endophenotypes for OCD may also benefit from including a broader range of disgust-relevant stimuli during conditioning procedures. Further, although the primary aim of the present study was to examine disgust learning as a potential OCD endophenotype, there are additional group comparisons that may inform conceptual models regarding disgust as a vulnerability factor. For example, comparisons of those with OCD to high and low disgust proneness subclinical OCD conditions may clarify a pattern of risk that is largely driven by disgust proneness.

Another important limitation of the present study is the reliance on self-report. Accordingly, future research employing more objective measures of conditioned disgust should be considered. One potential objective measure for consideration in future research is the levator labii activity, which has been shown to be a reliable indicator of disgust (Vrana, 1993). The tendency to avert one’s gaze or ‘oculomotor avoidance’ has also been shown to be a relatively specific disgust response that may compliment self-reported disgust in future research on disgust conditioning in OCD (Armstrong et al., 2014). Another study limitation is that the disgust conditioning task only probed differences in acquisition and extinction. Indeed, there may be other learning processes, such as disgust generalization (Berg et al., 2021; Olatunji and Tomarken, 2023), that may also be relevant to understanding OCD. Given that there is moderate evidence that OCD is associated with abnormal acquisition of conditioned fear responses and even stronger evidence of OCD’s association with impaired fear extinction processes (Cooper and Dunsmoor, 2021), delineating the extent to which OCD may be better explained by components of fear versus disgust conditioning is an important direction for future research. Given growing interest in identifying candidate psychological endophenotypes for OCD (e.g., Sica et al., 2016), future research addressing the present study limitations may provide more definitive data that can inform our understanding of the nature, function, and specificity of disgust learning in OCD.

## Figures and Tables

**Table 1:** Group symptom characteristics.

	OCD	Relatives	Controls	Minimum-Maximum	Test Statistic
OCI-R	31.90 (14.91)	11.00 (10.89)	8.92 (7.96)	0–59	*F* = 32.82, *p* < .001
DPSS-R	31.80 (10.65)	18.15 (11.84)	18.04 (6.98)	1–56	*F* = 17.41, *p* < .001
STAI	52.23 (10.18)	35.15 (11.90)	35.48 (10.06)	21–71	*F* = 23.62, *p* < .001
Y-BOCS	21.89 (6.76)	–	–	6–32	–

Note: OCD = obsessive-compulsive disorder group; Relatives = first-degree relative group; Controls = healthy control group. OCI-R = Obsessive-Compulsive Inventory-Revised; DPSS-R = Disgust Propensity and Sensitivity Scale-Revised; STAI = Spielberger State Trait Anxiety Inventory- Form Y; Y-BOCS = Yale-Brown Obsessive-Compulsive Scale.

**Table 2:** Means (SDs) for self-reported disgust responses to the CSs.

OCD Group				
CS Type	Stage			
	Habituation	Acquisition 1	Acquisition 2	Extinction
CS+	6.47 (1.42)	6.91 (0.80)	6.81 (1.16)	6.50 (1.26)
CS−	6.35 (0.91)	6.38 (1.14)	6.50 (0.76)	6.54 (0.65)
Relative Group				
CS type	Stage			
	Habituation	Acquisition 1	Acquisition 2	Extinction
CS+	6.17 (0.86)	6.14 (0.59)	6.38 (0.68)	6.19 (0.67)
CS−	6.23 (0.45)	6.10 (0.76)	5.98 (0.67)	6.02 (0.71)
HC Group				
CS type	Stage			
	Habituation	Acquisition 1	Acquisition 2	Extinction
CS+	6.14 (0.31)	6.40 (0.56)	6.48 (1.04)	6.24 (1.57)
CS−	6.22 (0.44)	6.08 (0.76)	6.22 (0.41)	6.18 (0.48)

Note. OCD Group = obsessive-compulsive disorder group; Relative group = first-degree relative group; HC Group = healthy control group. CS = conditioned stimulus; CS+ = food item paired with unconditioned stimulus (vomit video); CS− = food item not paired with unconditioned stimulus.

## Footnotes

1 The post-baccalaureate research assistant (RA) underwent approximately 10–15 h of training on the MINI (i.e., review of rationale/structure of interview, observing MINIs conducted by doctoral-level graduate students) and was supervised by a licensed clinical psychologist. After training, the first 3–5 MINIs conducted by the RA were observed by a doctoral-level graduate student and the RA met weekly with the graduate students and licensed clinical psychologist to resolve issues related to diagnostic clarity.
